# A MODEL FOR DISSEMINATING EVIDENCE-BASED RESIDENT-TO-RESIDENT AGGRESSION INTERVENTION PROGRAMS

**DOI:** 10.1093/geroni/igad104.0150

**Published:** 2023-12-21

**Authors:** Karl Pillemer, Matthew Luebke

**Affiliations:** Cornell University, Ithaca, New York, United States; Cornell University, Ithaca, New York, United States

## Abstract

In elder mistreatment research, a key problem is the failure to disseminate interventions to broad audiences. This gap prevents most interventions from benefitting victims and improving their quality of life. Interventions are often not disseminated because of a lack funding, staffing, and support from provider organizations. We report on a novel, low-cost dissemination model to circumvent these barriers and expand program reach. The program, Improving Resident Relationships in Long Term Care (IRRL), trains staff to recognize resident-to-resident aggression (RRA) and to learn how to manage it. The training consists of two sessions, (1) recognition and (2) management. A dissemination model was created that included systematic marketing, outreach, and organizational sponsorship. We describe the model, which is replicable for a variety of dissemination efforts: 1) creating an easily accessible manual, supporting documents, and evaluation materials; 2) designing a user-friendly website for distribution; 3) identifying provider associations for program marketing; 4) developing partnerships with LTC organizations for dissemination; 5) providing national informational webinars; 6) offering webinar participants subsequent train-the-trainer sessions and coaching. This plan is feasible with limited expenditures and can be accomplished via the use of student research assistants. Using this model, the IRRL program has gained organizational support and has employed web-based training to reach over 300 providers. The dissemination of the IRRL program offers a potential method for intervention delivery with limited funding. Implications for future dissemination research are discussed.

